# Enhancing diagnosis of T-cell lymphoma using non-recombined T-cell receptor sequences

**DOI:** 10.3389/fonc.2022.1014132

**Published:** 2022-12-08

**Authors:** Yi-Lin Chen, Chung-Liang Ho, Chen-Yan Hung, Wan-Li Chen, Chen Chang, Yi-Hsin Hou, Jian-Rong Chen, Pin-Jun Chen, Nan-Haw Chow, Wenya Huang, Ya-Ting Hsu, Tsai-Yun Chen, Tsunglin Liu

**Affiliations:** ^1^ Department of Pathology, National Cheng Kung University Hospital, Tainan, Taiwan; ^2^ Molecular Medicine Core Laboratory, Research Center of Clinical Medicine, National Cheng Kung University Hospital, Tainan, Taiwan; ^3^ Medical Laboratory Science and Biotechnology, College of Medicine, National Cheng Kung University, Tainan, Taiwan; ^4^ Institute of Molecular Medicine, College of Medicine, National Cheng Kung University, Tainan, Taiwan; ^5^ Department of Biotechnology and Bioindustry Sciences, National Cheng Kung University, Tainan, Taiwan; ^6^ Section of Hematology/Oncology, Department of Internal Medicine, National Cheng Kung University Hospital, Tainan, Taiwan

**Keywords:** T-cell lymphoma, biomarker, clonality test, BIOMED-2, T-cell receptor (TCR), non-recombined TCR sequence

## Abstract

Clonality assessment, which can detect neoplastic T cells by identifying the uniquely recombined T-cell receptor (TCR) genes, provides important support in the diagnosis of T-cell lymphoma (TCL). BIOMED-2 is the gold standard clonality assay and has proven to be effective in European TCL patients. However, we failed to prove its sensitivity in Taiwanese TCL patients, especially based on the TCRβ gene. To explore potential impact of genetic background in the BIOMED-2 test, we analyzed TCRβ sequences of 21 healthy individuals and two TCL patients. This analysis suggests that genetic variations in the BIOMED-2 primer sites could not explain the difference in sensitivity. The BIOMED-2 test results of the two TCL patients were positive and negative, respectively. Interestingly, a higher percentage (>81%) of non-recombined TCRβ sequences was observed in the test-negative patient than those of the test-positive patient and all healthy individuals (13~66%). The result suggests a new TCR target for enhancing TCL diagnosis. To further explore the hypothesis, we proposed a cost-effective digital PCR assay that quantifies the relative abundance of non-recombined TCRβ sequences containing a J2-2P~J2-3 segment. With the digital PCR assay, bone marrow specimens from TCL patients (n=9) showed a positive outcome (i.e., the relative abundance of the J2-2P~J2-3 sequences ≧5%), whereas non-TCL patients (n=6) gave a negative result. As five of nine TCL patients had a negative BIOMED-2 test result, the J2-2P~J2-3 sequences may improve TCL detection. This is the first report showing the capability of characterizing non-recombined TCR sequences as a supplementary strategy for the BIOMED-2 clonality test.

## Introduction

T-cell lymphoma (TCL) is characterized by the uncontrolled expansion of one or more malignant T cells, that is, malignant T-cell clones. TCL diagnosis is based on multiple parameters including clinical features, morphology, immunophenotype of blood cells and/or bone marrow (BM) biopsy, and genetic alterations such as translocation (1, 2). Because TCL has multiple subtypes (3, 4) and some TCLs have overlapping features, the diagnosis is complex in a small proportion of cases (5). In addition, as small biopsies are becoming popular, it is getting more difficult to distinguish neoplastic T cells from reactive T-cell infiltrates in the morphological tests (6). Given these difficulties, clonality assessment is now widely used to assist TCL diagnosis (7).

In clonality assessment, distinct T cells are identified by their unique recombination of T-cell receptor (TCR) genes. The current gold standard clonality test for lymphoma is a multiplex polymerase chain reaction (PCR)-based assay using BIOMED-2 primers, which were developed by the EuroClonality consortium (8, 9). After PCR amplification, a heteroduplex analysis or GeneScanning is conducted to distinguish monoclonal from polyclonal products, the former of which often suggests the diagnosis of lymphoma (7, 10).

The BIOMED-2 test has proven to be powerful in a European cohort (11), where 94% of all TCL cases showed TCR clonality. However, their performance depends on TCL subtypes. For example, TCR clonality was detected in all cases of peripheral TCL, not otherwise specified (PTCL-NOS) and 95% cases of angioimmunoblastic T-cell lymphoma (AITL). In contrast, only 79% of anaplastic large cell lymphoma (ALCL) cases showed TCR clonality. The overall sensitivity of the BIOMED-2 test in Asian cohorts was less satisfactory. In Korean and Taiwanese cohorts, TCR clonality was detected in 83% and 76% of TCL cases, respectively (12, 13). In the PTCL-NOS cases, the detection rates were 90% and 85% in the Korean and Taiwan cohorts, respectively, which were lower than the 100% rate in the European cohort.

In the BIOMED-2 test, TCR clonality was inferred based on the combined results of TCRβ, TCRγ, and TCRδ genes, the first two of which covered the most cases (11, 14). In European cases of PTCL-NOS, the detection rate based only on TCRβ or TCRγ (98% or 94%, respectively) was slightly lower than the combined rate (100%). In the Korean cases of PTCL-NOS, however, only 46% and 51% showed TCRβ and TCRγ clonality, respectively, which was approximately half of the combined rate (90%). In the Taiwanese cases of PTCL-NOS, the detection rate of TCRβ clonality (54%) was lower than that of TCRγ (85%). This suggests a role for genetic background in the BIOMED-2 test results, which has not been explored previously, to the best of our knowledge. In addition, TCL diagnosis in Asian patients, especially based on the TCRβ gene, still needs improvement.

A new TCR target may be helpful for enhancing the sensitivity of TCL diagnosis. The BIOMED-2 primers target the V and J gene segments to detect various completely recombined TCR alleles (8). For the TCRβ and TCRδ genes, BIOMED-2 includes primers that target upstream of the D gene segments to detect partially recombined DJ alleles. Non-recombined (NR) TCR sequences, which contain only a J gene segment spliced to a constant (C) segment, have also been observed in the next-generation sequencing (NGS) data of cDNAs prepared *via* a 5′ rapid amplification of cDNA ends (RACE) approach (15, 16). We note that 5′ RACE amplifies RNA, whereas the BIOMED-2 test amplifies DNA. In both studies, NR TCRβ sequences comprised more than half of the TCRβ data. NR TCR sequences have been largely overlooked in literature and their potential to enhance TCL diagnosis is never studied.

Here, we investigated whether genetic background can explain the reduced sensitivity of the BIOMED-2 test in Taiwanese individuals. We recruited 21 healthy individuals and two TCL patients whose BIOMED-2 test results were positive and negative, respectively, and sequenced their TCRβ transcripts amplified using 5’ RACE. This analysis suggested that genetic variations in the BIOMED-2 primer sites could not explain the reduced sensitivity. Interestingly, the TCRβ repertoire analysis revealed a biomarker potential of NR TCR sequences in TCL diagnosis. The biomarker potential was further confirmed using a digital PCR assay on nine TCL and six non-TCL patients. This is the first study to characterize NR TCR sequences in detail and show their application in TCL diagnosis.

## Materials and methods

### Subject recruitment

To explore TCRβ genetics and repertoire, we recruited 21 healthy individuals (defined as one without any known serious illness) and two TCL patients at the National Cheng Kung University Hospital (NCKUH) in 2011. For the digital PCR assay, we recruited TCL patients visiting the outpatient clinic at NCKUH between June 2018 and February 2019. Only the TCL patients whose BM biopsy was confirmed to have infiltrated malignant T cells were included. In addition to TCL patients, we recruited non-TCL patients with blood disorders as controls.

### TCL diagnosis

All lymphoma cases were confirmed by BM findings and were correlated with clinical features. Immediately after the collection of BM aspirate and biopsy, the imprint smears were independently examined for morphological details by two pathologists. Only the samples confirmed to have infiltrated malignant T cells were subjected to molecular tests, including cytomorphology evaluation, immunophenotyping, and cytogenetic analysis. A review of the pathology records and clinical history was performed by the pathologist (CC) and the hematologists (YTH and TYC). Corresponding immunohistochemical and clonality studies were also reviewed.

### BM sample preparation

For each patient with a BM or blood disorder, part of the BM aspirate was subjected to molecular diagnoses, including morphological evaluation and immunophenotyping. The remaining samples were stored in liquid nitrogen and those with approved by the local ethics committee were included in this study. For digital PCR, we thawed the stored BM samples and extracted total RNA from mononuclear cells using QIAamp RNA Blood Mini Kit (Qiagen, Hilden, Germany) following manufacturer’s instructions. For quality control, two primer pairs were used to amplify the *GAPDH* (165 bp; forward primer: GAAGGTGAAGGTCGGAGTC, reverse primer: TGGAATTTGCCATGGGTGGA) and *2-microglobulin* (256 bp; forward primer: TGGAGGCTA TCCAGCGTACT, reverse primer: CGGCAGGCATACTCATCTTT) genes from 100 ng of each sample, as previously described (17). Qualified samples (165/256 bp) were selected for digital PCR. Another portion of the BM aspirate was subjected to the BIOMED-2 test and a DNA quality of 400 bp was required.

### BIOMED-2 clonality test

The BIOMED-2 test was implemented based on the earlier standardization of BIOMED-2 Ig/TCR clonality protocols1 (9) and laboratory validation2 (12). For the TCRβ and TCRγ genes, each reaction included a multiplex PCR assay that amplified the V (or D) to the J regions of the TCR gene. Both monoclonal and polyclonal controls with the expected size ranges were used as positive and negative controls, respectively. Multiplex PCR and heteroduplex analyses were interpreted according to the latest EuroClonality/BIOMED-2 guidelines (9). Unless otherwise specified, the BIOMED-2 test was performed on BM samples.

### 5’ RACE and NGS

To inspect the TCRβ genetics and repertoire, we collected peripheral blood (PB) and extracted total RNA from mononuclear cells using Ficoll-Paque (Ficoll-Paque™ PLUS) density gradient centrifugation. TCRβ sequences in the RNA samples were amplified using a 5’ RACE approach, described in our previous work (16). Briefly, RNAs of TCRβ genes were converted into cDNAs using a gene-specific primer GSC1 (CACGTGGTCGGGGWAGAAGC). First PCR was performed using another gene-specific primer GSC2 and the 5’ universal primer (UPM) with an overhang (GSC2: GGGTGGGAACACCTTGTTCAGGT; UPM: CTAATACGACTCACTATAGGGC, overhang: CTAATACGACTCACTATAGGGC). The UPM served as a forward primer and the overhang served as a primer site for nested PCR. In the nested PCR, an equimolar of three primers (adaptor1-UPM, adaptor2-TCRβ-C1/2) (adaptor1: CGTATCGCCTCCCTCGCGCCATCAG; adaptor2: CTATGCGCCTTGCCAGCCCGCTCAG; TCRβ-C1: GGGTGGGAACACCTTGTTCAGGT; TCRβ-C2: GGGTGGGAACACCTTTTTCAGGT) was added. The adaptor sequences were for emulsion PCR in 454 sequencing. Each adaptor also contained a 10 bp barcode for labeling sample. Labeled samples were pooled together for eight runs of 454 sequencing on Genome Sequencer FLX using a GS Lib-L emPCR kit (Roche Applied Science) following manufacturer’s protocol.

### Sequence analysis

Raw 454 reads were de-multiplexed and trimmed to remove primers and barcodes, described in (16). Specifically, we concatenated the technical sequences and aligned them to raw reads using BLAST (v2.2.31+; options: -word_size 4 -gapopen 1 -gapextend 1 -evalue 0.01). For each raw read, the best aligned barcodes at both read ends were identified. Reads with inconsistent barcodes at the two ends were discarded. We then trimmed technical sequences from the reads and discarded short (<150 bp) trimmed reads. This ensures that intronic segments in non-recombined TCRβ sequences could be detected. The processed reads of each sample in different sequencing runs were combined for VDJ annotation.

We used TRIg (16) to annotate the processed reads, and modified the results to (1) merge broken alignments, (2) allow interchange between two C segments of the TCRβ gene, and (3) adjust alignments at splicing sites. A broken alignment was referred to as two aligned segments that were close in both the read and the TCRβ gene. The two read segments likely came from the consecutive segment in the TCRβ gene and was broken because of low-quality bases in-between. We checked all neighboring alignments and calculated distances in the read and the TCRβ gene. When the two distances differed by less than half of the larger distance, the two alignments was merged. In the second modification, we inspected the order of read segments. In the TCRβ gene, the gene segments are in the order of V, D1, J1, C1, D2, J2, and C2. We expected the segments in a read to follow the same order, e.g., V-J2-C2 was ordered while V-J2-C1 was not. Because the C1 and C2 gene segments were highly similar (only one base different in 23 bases at the 5’ RACE primer site), a sequencing error could result in a different best alignment. Therefore, when the C1 segment was not ordered in a read (e.g., V-J2-C1), we replaced the C1 annotation with C2. In the third modification, when two neighboring alignments overlapped in the read, we split the overlapping segment at a potential splicing site, defined as a locus after and before which GT and AG were present in the TCRβ gene, respectively.

Throughout this work, the TCRβ reference was referred to as the reference sequence in TRIg. For predicting splicing site, we used NetGene2 webserver (v2.42) (18). Recombination signal sequence (RSS) sites in the TCRβ reference were predicted using the RSSsite webserver (19).

### Statistical analysis

Principal component analysis was implemented using the function fiz_pca_ind() in the factoextra library (v1.0.7) of R package (v4.1.3). The relative abundances of the J2-2P~J2-3 sequences in TCL and non-TCL patients were compared by Wilcoxon rank test using stat_compare_means() in the library ggpubr (v0.4.0) of R package (v4.1.3).

### Genetic variants

To detect single nucleotide polymorphisms (SNPs), the aligned segments by TRIg were re-aligned to the TCRβ reference using bwa (v0.7.17; command bwasw) (20). The alignments were sorted by reference coordinate using samtools (v1.7) (21), and converted into a pileup file (command and options: mpileup -B -q 1 -f tcrb.fa). The pileup file was analyzed by VarScan (v2.4.4) (22) to call SNPs (command and options: pileup2snp –min-coverage 20 –min-var-freq 0.2 –p-value 0.05).

To locate the BIOMED-2 primer sites, primer sequences of the TCRβ gene in van Dongen et al. (8) were aligned to the TCRβ reference using USEARCH (v11.0.667; command search_oligodb) (23) allowing at most four mismatches. For each gene segment, the best aligned primer was selected to infer the binding site. We identified primer sites in all 49 functional V and 13 J gene segments except V17. For each locus in the primer sites, we searched the NCBI dbSNP database (24) for variant information in the Asian population. Because the TCRβ reference differed slightly from the NCBI record, the coordinate mappings were obtained *via* BLAT alignments (v4.0.1; default options) (25). SNPs in a few V gene segments (V3-2, V4-3, V5-8, V6-3, V6-9, and V7-8) were excluded. We discarded SNPs if the allele frequencies in three Asian populations (Asian, East Asian, and Other Asian) were zero. The highest frequencies in the three populations were shown.

### Digital PCR

Primers and probes for the Clarity™ digital PCR system (J2-2P forward primer: 5’-AGGCGCTGCTGGGCGTCT-3’ and reverse primers: 5’-GGGTGGGAACACGTTTTTCAGG-3’, J2-2P probe: 5’-HEX-CTCTCCCAG/ZENCACCCAGAACCAGGA/3IABkFQ/-3’; J2-3 forward primer 5’-GCACAGATACGCAGTATTTTGG-3’ and reverse primers: 5’-TCAGCTCCACGTGGTCGGGGT-3’, J2-3 probe: 5’-FAM-CTGACAGTG/ZEN CTCGAGGACCTGAAAAACGT/3IABkFQ/-3’) were synthesized by Integrated DNA Technologies. Each reaction mix consisted of 1× LightCycler TagMan Master Reaction Mix (Roche), 1× Clarity™ JN solution, 0.66 μM forward and reverse primers, 0.53 μM probe, 1 μL cDNA sample and PCR grade water top up to 15 μL. Using the Clarity™ auto loader, the mixture was then delivered onto the chip, where it was sub-divided into 10,000 partitions. The partitions were then sealed with the Clarity™ Sealing Enhancer using 245 μL Clarity™ sealing fluid, followed by VWR^®^ thermal cycler with the following parameters: initial cycle of 95°C for 5 min and 40 cycles of 95°C for 50 s and of 55°C for 90 s, and 70°C for 5 min, 4°C hold. Ramp rate was set to 1°C/s. After PCR amplification, the tube strips were transferred to the Clarity™ reader, which detected fluorescent signals of HEX and FAM. The data were analyzed by the Clarity™ software (version 2.1), and a proprietary algorithm was used for setting each threshold based on fluorescent intensities to determine the proportion of positive partitions out of the total. Based on this information, the software determined the DNA copies per microliter of dPCR mix using the Poisson statistics.

## Results

### Study design

To explore the genetic background of Taiwanese individuals in clonality assessment, we recruited 21 individuals without TCL or any known serious illness and two TCL patients (**Supplementary Table 1**). It is to be noted that one individual (I4) was pregnant and another one (I15) had a cold at the time of sample collection. Of the two TCL patients, one was diagnosed with ALCL (I11) and the other with AITL (I13), respectively. The diseases of both TCL patients were in stage IVb, as determined according to cell morphological analysis and immunohistochemical findings. The BIOMED-2 test result based on TCRβ and TCRγ genes was positive for the AITL patient, and negative for the ALCL patient (**Supplementary Figure 1**).

To investigate TCRβ genetics, we collected PBs from all 23 individuals for TCRβ sequencing. To check the consistency between PB and BM, a part of the BM sample of the ALCL patient (I11b; I11a was the PB sample) was also subjected to TCRβ sequencing. To sequence the TCRβ transcripts, we extracted total RNA from each of the 24 PB samples and the TCRβ sequences were amplified using 5’ RACE. Note that the 5’ RACE approach amplifies RNA sequences and their consensus was used to represent the corresponding DNA sequence. The 454 sequencing generated 380164 reads. We trimmed technical sequences from the reads and discarded short (<150 bp) trimmed reads. Of the resulting processed reads, 101903 were VDJ annotated for further analysis (**Supplementary Table 2**). The data also allowed us to examine TCRβ repertoire, that is, the composition of various types of recombined and non-recombined TCRβ sequences.

### SNPs in the TCRβ gene

A plausible explanation for the lower sensitivity of the BIOMED-2 test in Asian TCL patients was the less optimized BIOMED-2 primers for Asians because the primers were originally tested in Europe. To examine this possibility, we detected SNPs in 21 healthy individuals and two TCL patients.

We identified 44 distinct SNPs in all individuals (**Supplementary Table 3**). Approximately half (21) of the distinct SNPs were also reported in a previous study (26), in which the SNPs were found based on sequencing data of four populations, including Chinese. This indicates the accuracy of our SNP detection method. The ALCL patient had three SNPs and one SNP in the V and J gene segments, respectively; however, none of them were in the BIOMED-2 primer sites. The AITL patient had 12 SNPs and one SNP in the V and J gene segments, respectively, and one SNP was located in the primer binding site of the V10-3 gene segment (**Supplementary Table 3**). This SNP resulted in a slightly better match with the primer (**Supplementary Table 4**). However, the site was near the 5’ end; thus, we did not expect a notable enhancement in the primer efficiency. For all other individuals, only three showed this SNP in V10-3, and no SNP was found in any other primer site.

To expand the SNP coverage on the TCRβ gene, we searched the NCBI dbSNP database for variants at the primer sites. A total of 21 SNPs, including the SNP in V10-3, were found at the primer sites (**Supplementary Table 5**). However, only five (V10-2, V7-4, V7-6, V11-3, and V28) were within 10 bases from the 3’ end, and only one (V11-3) resulted in a better match. The variant allele frequencies were relatively low (V10-2:0.0004, V7-4:0.018, V28:0.002) except for V7-6 (0.5). In our data, the usages of the V7-6, V10-2, or V7-4 gene segments were below 3% for all individuals. This suggests that genetic background could not explain the reported lower sensitivity of the BIOMED-2 test in Taiwanese TCL patients.

### Characterization of TCRβ sequences

**Figure 1:**
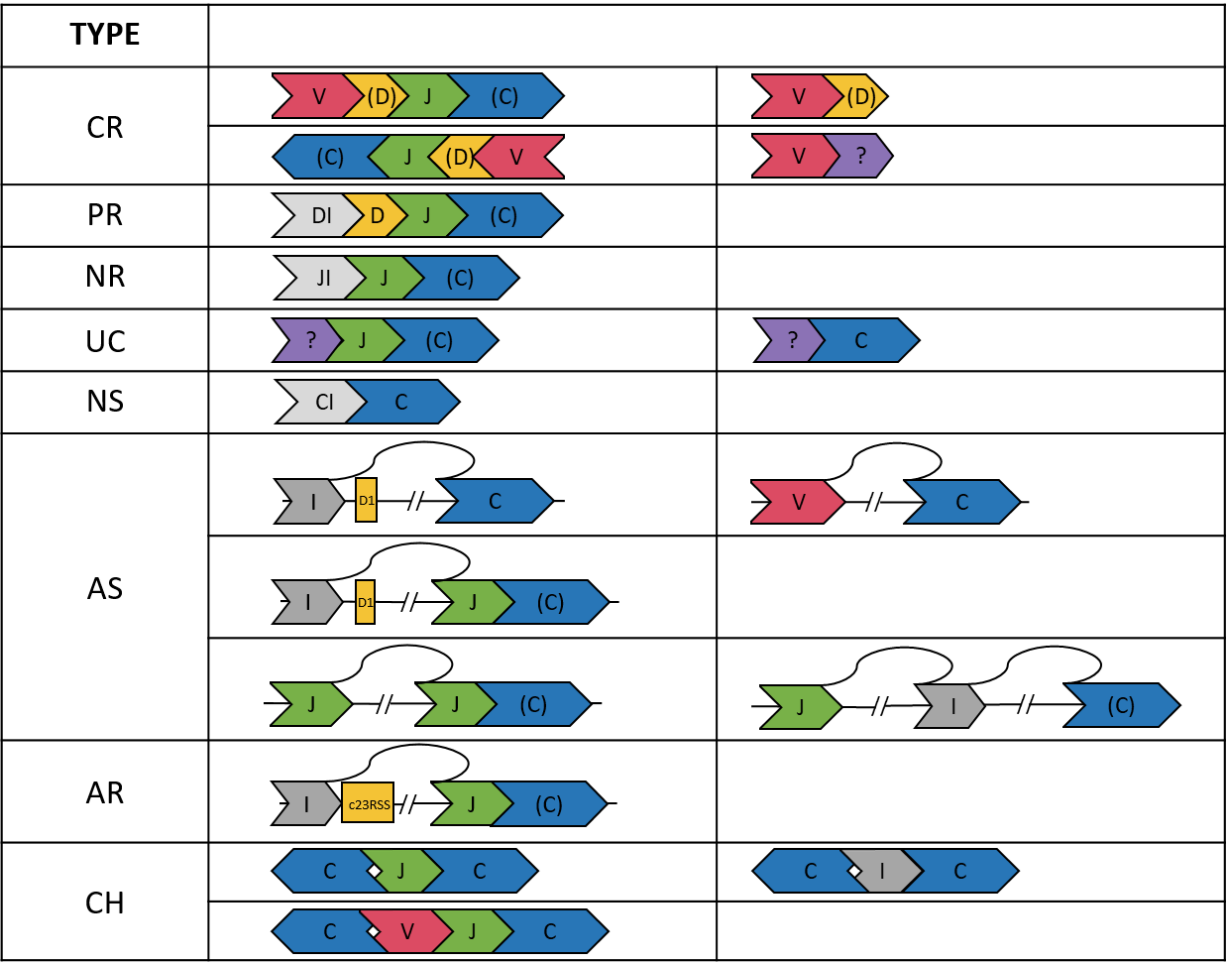
Illustration of the TCRβ sequences in the eight categories. A segment in “()”, e.g., (D, C), may be absent. The DI, JI, and CI segments indicate introns upstream of the D, J, and C genes, respectively. The “?” segment is unaligned. Arrows indicate strands of the sequences.

Based on the VDJ annotation, we classified 101903 reads into eight categories: completely recombined (CR), partially recombined (PR), NR, non-spliced (NS), aberrantly spliced (AS), aberrantly recombined (AR), uncertain (UC), and chimeric (CH). The definitions were described below and shown in **Figure 1**.

#### CR reads

We considered a read CR if it was composed of ordered V-(D)-J-(C) segments (D and C optional) on either the plus or minus strand. The requirement of being ordered on the same strand applied to all categories except CH. We also found 8315 reads composed of only V-(D) segments. Most (91.3%) of these reads ended within the V gene segment or had a ≦60 bp unaligned segment at the end, which might be a CDR3 sequence. Thus, the V-(D) reads were likely not long enough to cover the J segments. Therefore, V-(D) reads were also considered CR, but were excluded from the statistics of VJ pairings and CDR3 clonotypes.

#### PR reads

A D-J-(C) read was considered PR. In most (98.6%) of the D-J-(C) reads, the D segment contained at least 15 bp intron in the immediate upstream of the D segment. This ensured that the D gene was not recombined to a V gene. We also found three reads comprising only an annotated D segment. These reads had an unaligned segment at the end, which might be the D-J junction segment containing random nucleotides. Thus, the D-only reads were also considered PR, but were excluded from the D-J statistics.

#### NR reads

We considered a J-(C) read NR. Most (99.4%) of the J-(C) reads contained a ≧15 bp intronic segment in the immediate upstream of the J segment, which ensured the non-recombination. For the remaining J-(C) reads, most (98.1%) had a ≧30 bp unaligned segment in the upstream of the J segment, which might be the CDR3 or D-J junction segment. J-(C) reads without the ≧15 bp upstream intron were considered UC because the recombination status was not certain.

#### NS reads

An NS read was composed of only a C segment. The absence of splicing was again ensured by a ≧15 bp intron upstream the C gene segment for most (93.9%) C-only reads. The remaining C-only reads had a long (>120 bp) unaligned segment and were considered UC because the splicing status and identity of the unaligned segment were not clear.

#### AS reads

When a C segment was concatenated to a V, D, or intergenic (I) segment in the upstream, the read was considered AS. To justify this classification, we first inspected I-C reads and identified the splicing points between the intergenic and the C segments. Among the I-C reads with an identified splicing point, most (99.4%) showed a splice donor sequence GT immediately after the intergenic segment in the TCRβ gene. Interestingly, the majority (80.0%) of the splicing sites were at position 548538 in the TCRβ reference, which was 174 bp upstream the D1 gene segment. This locus was among the top ten predicted splicing donor sites across the TCRβ reference (**Supplementary Table 6**). Aberrant splicing in other configurations (e.g., in V-C and I-J-(C) reads) were examined, and we found splice donor and receptor sequences in most reads (e.g., 91.7% in V-C reads and 81.6% in I-J-(C) reads).

#### AR reads

An I-J-(C) read might be derived from aberrant recombination to a cryptic RSS site. In immune receptor recombination, there are two types of RSSs: 12RSS and 23RSS. During DJ recombination, the 12RSS at 5’ end of a J gene segment pairs with the 23RSS at 3’ end of a D gene segment. The former may also pair with a cryptic 23RSS on the plus strand in an intergenic region, resulting in an I-J-(C) read. We found only three I-J-(C) read whose intergenic segment ended within 30 bp from the start of a cryptic 23RSS site. This indicates aberrant recombination was a rare event in our samples.

#### CH reads

A read was considered CH if (1) it contained segments on different strands of the TCRβ gene, (2) the segments were not strictly increasing or decreasing in the TCRβ gene when the read was on the plus or minus strand, respectively, and (3) it contained two or more C segments (e.g., C-J-C and C-I-C reads).

CH and UC reads accounted for 3% of the data (**Supplementary Table 2**) and were excluded from the following statistics.

### Clonality based on CR reads in the TCRβ repertoire

**Figure 2:**
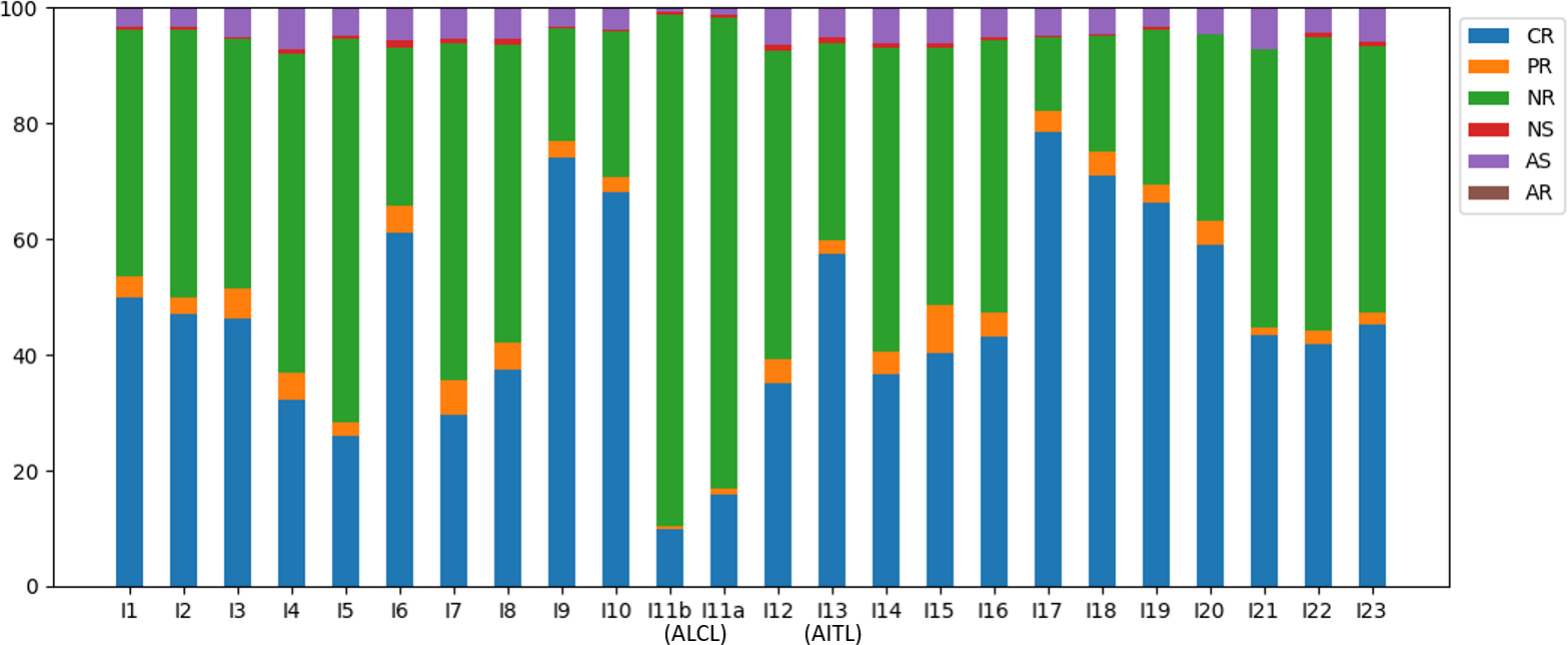
Compositions of the TCRβ reads in the six categories across all samples.

**Figure 3:**
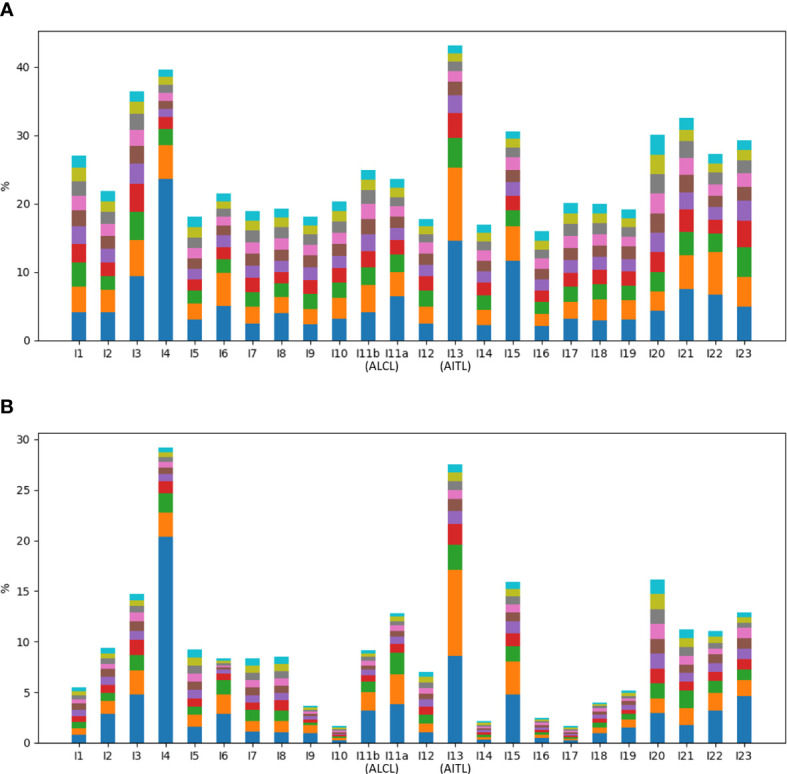
Percentages of top ten abundant **(A)** VJ pairings and **(B)** CDR3 clonotypes in all samples.

Of all the annotated reads, 41.0% were CR, and the percentages ranged 9.9-78.4% across all samples (**Figure 2**). To examine clonality, we obtained the compositions of VJ pairings and CDR3 clonotypes in the CR reads for all samples. Samples I4, I13, and I15 showed the three highest percentages of the top abundant VJ pairings (**Figure 3A**). This could be explained by the pregnancy of individual I4 and cold of I15 at the time of sample collection. Sample I13 was from the AITL patient, and its dominance of the most abundant VJ pairing was consistent with the positive result of the BIOMED-2 test. Similar observations were made in terms of CDR3 clonotypes, and the dominance of the top two clonotypes was even more apparent for I4 and I13 than for other individuals (**Figure 3B**).

The absence of a dominant clonotype in the ALCL samples was consistent with the patient’s negative result of the BIOMED-2 test. Thus, we confirmed that the BIOMED-2 test failed to identify the ALCL patient because no clonal TCRβ existed in the samples. Note that the top VJ pairings in samples of the two TCL patients were not V10-3, which supports our hypothesis that genetic background did not explain the negative BIOMED-2 test result. Although no clonality was observed for the ALCL patient, the two samples showed the highest percentages of NR reads (88.3% and 81.3% in I11b and I11a, respectively) compared to other samples (12.8-66.3%) (**Figure 2**). This motivated us to explore the biomarker potential of NR reads.

### Compositions of NR reads

**Figure 4:**
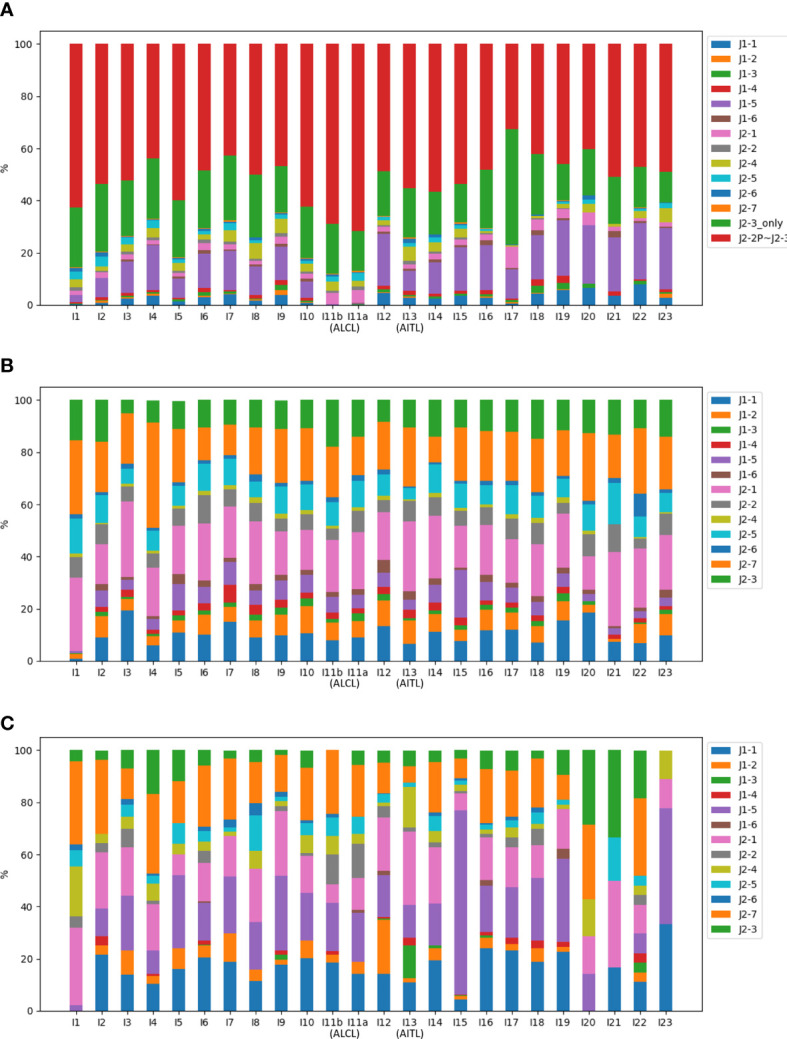
Compositions of the J gene segments in **(A)** NR, **(B)** CR, and **(C)** PR reads of all samples. In **(A)**, the J2-3 reads are divided into J2-2P~J2-3 and J2-3_only reads.

The NR reads were further classified based on the J gene segment spliced into the C gene segment. Among the 13 J gene segments, J2-3 was the most abundant across all samples (**Figure 4A**). Recall that all the J segments extended into the upstream intron, and we found that a majority (76.2%) of the J2-3 segments covered the upstream pseudogene J2-2P, defined as J2-2P~J2-3 sequences. Therefore, the J2-3 reads were further split into two categories: J2-2P~J2-3 and J2-3_only. The percentage of the J2-2P~J2-3 reads was the highest in the two ALCL samples (69.0% and 71.6% in I11b and I11a respectively) compared to those in the rest (32.7-62.5%). Thus, the J2-2P~J2-3 sequences could be a molecular target for clinical TCL diagnosis.

**Figure 5:**
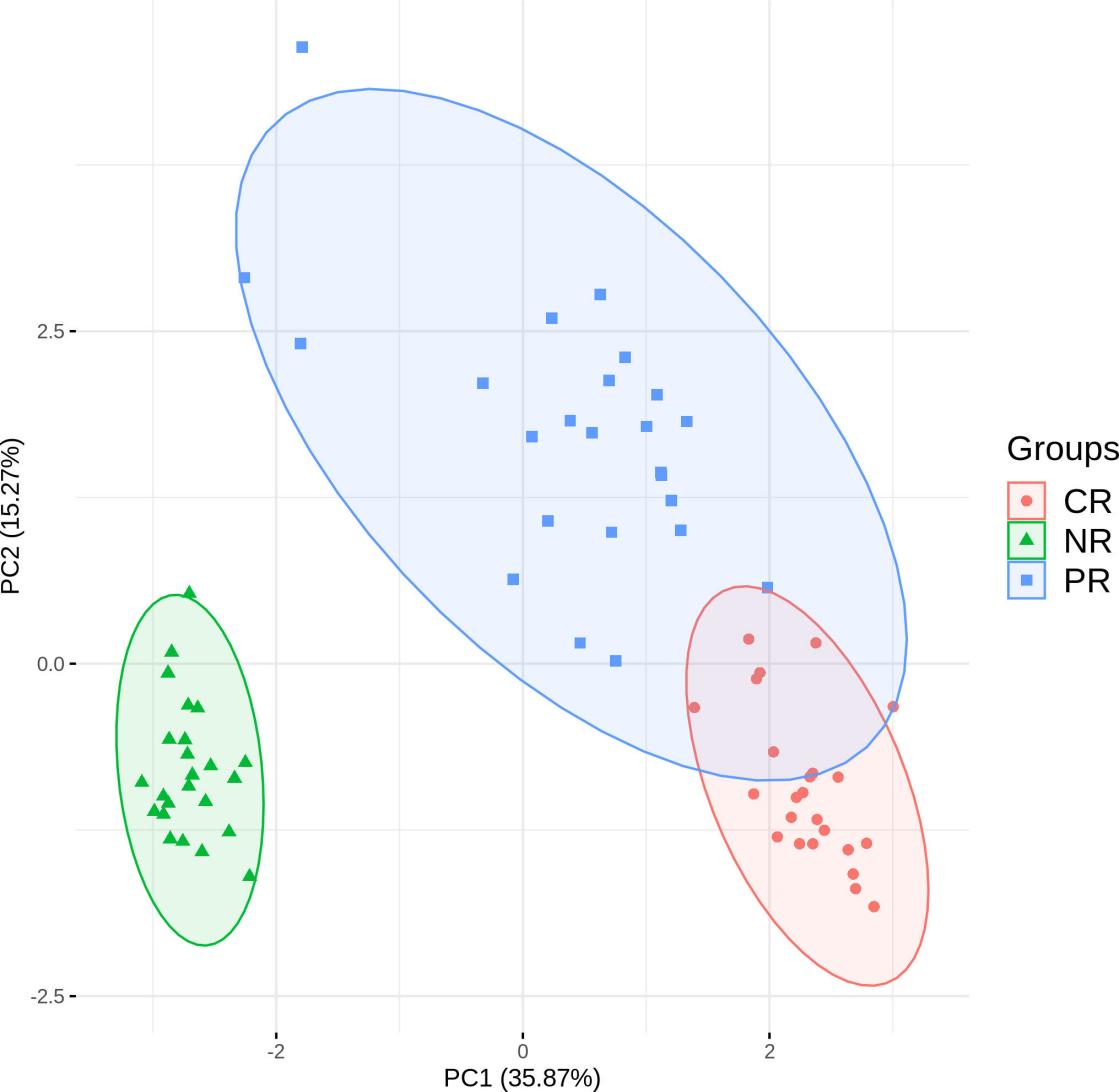
Principal component analysis based on J gene usage in CR, PR, and NR reads.

**Figure 4** shows CR and PR reads had similar compositions of J gene segments, which were clearly distinct from the NR reads. The relationship was even more apparent in the principal component analysis (**Figure 5**). Moreover, CR and PR reads were distributed to different J genes more uniformly compared to the NR reads (**Supplementary Figure 2**). Thus, NR sequences were likely generated using a different mechanism from the CR and PR sequences. We also note that the PB and BM samples of the ALCL patient were highly similar in many aspects (**Figures 2–4**). This suggests that PB and BM samples of the same individual would provide a similar signal.

### Clinical assessment of the biomarker potential of J2-2P~J2-3 reads

Because NGS is still relatively expensive, clonality assessment using qPCR or digital PCR is more practical in the clinic. For digital PCR, we designed primers targeting the J2-2P and C region of the TCRβ gene for amplifying the J2-2P~J2-3 sequences and validated that the primers amplified the desired segment using Sanger sequencing (**Supplementary Figure 3**). Note that digital PCR amplifies RNA sequences of TCRβ genes. In addition, we designed a J2-3 primer for probing CR, PR, and NR sequences containing a J2-3 segment. Comparison of the two quantities in digital PCR indicated the relative abundance of NR sequences.

For clinical assessment, we recruited nine TCL patients and six non-TCL patients (**Table 1**). Seven TCL patients were diagnosed with PTCL-NOS. From each patient, the BM aspirate was extracted and subjected to both the BIOMED-2 test and digital PCR. Five of nine TCL patients had a negative BIOMED-2 test result (**Table 2**). Using digital PCR, we obtained the concentrations of the J2-2P~J2-3 sequences and sequences containing a J2-3 gene segment in the BM samples and calculated the ratio (**Table 2**). The concentrations and ratio might be altered by non-T cells in the BM sample if they expressed the J2-2P~J2-3 and the J2-3 transcripts. To address the concern, we detected the two types of transcripts in nine non-T cell lines and two TCL cell lines. The J2-2P~J2-3 and the J2-3 transcripts were not detected in any of the non-T cell lines (**Supplementary Figure 4**). In contrast, the transcripts were detected in the two TCL cell lines. Therefore, we expected non-T cells would not alter the quantification of the J2-2P~J2-3 and the J2-3 sequences.

**Table 1:** Characteristics of TCL and non-TCL patients for digital PCR.

Individual	Age	Gender	Disease
TCL1	67	M	PTCL-NOS
TCL2	76	F	PTCL-NOS
TCL3	66	M	PTCL-NOS
TCL4	63	F	PTCL-NOS
TCL5	37	M	PTCL-NOS
TCL6	74	F	PTCL-NOS
TCL7	67	M	PTCL-NOS
TCL8	14	M	T cell acute lymphoblastic leukemia
TCL9	62	M	Subcutaneous panniculities-like T-cell lymphoma
Non-TCL1	61	M	Megakaryocytic hyperplasia compatible with idiopathic thrombocytopenic purpura
Non-TCL2	47	F	Anemia
Non-TCL3	32	M	Acute Promyelocytic Leukemia
Non-TCL4	62	F	Chronic Myeloid Leukemia
Non-TCL5	32	M	Acute Monoblastic Leukemia
Non-TCL6	83	F	Chronic Myelomonocytic Leukemia

**Table 2:** Concentrations of the J2-2P~J2-3 and the J2-3 sequences measured by digital PCR and the ratios (J2-2P~J2-3 to J2-3) for TCL and non-TCL patients.

Individual	J2-2P~J2-3 (copies/ul)	J2-3 (copies/ul)	ratio (%)	BIOMED-2 test
TCL1	2.6	9.7	26.6	TCRγ: monoclonal; TCRβ: polyclonal
TCL2	11.5	83.3	13.8	TCRγ: monoclonal; TCRβ: polyclonal
TCL3	5.0	50.6	10.0	TCRγ: monoclonal; TCRβ: polyclonal
TCL4	4.9	21.9	22.5	TCRγ: monoclonal; TCRβ: polyclonal
TCL5	8.2	16.8	48.7	TCRγ: polyclonal; TCRβ: polyclonal
TCL6	1.0	3.1	31.1	TCRγ: polyclonal; TCRβ: polyclonal
TCL7	12.7	225.6	5.6	TCRγ: polyclonal; TCRβ: polyclonal
TCL8	23.0	261.8	8.8	TCRγ: polyclonal; TCRβ: polyclonal
TCL9	2.6	30.5	8.6	TCRγ: polyclonal; TCRβ: polyclonal
Non-TCL1	8.6	398.0	2.2	N.A.
Non-TCL2	8.2	405.5	2.0	N.A.
Non-TCL3	3.3	158.3	2.1	N.A.
Non-TCL4	33.8	793.8	4.3	N.A.
Non-TCL5	2.6	79.3	3.3	N.A.
Non-TCL6	2.8	80.6	3.6	N.A.

NA, Not applicable.

**Figure 6:**
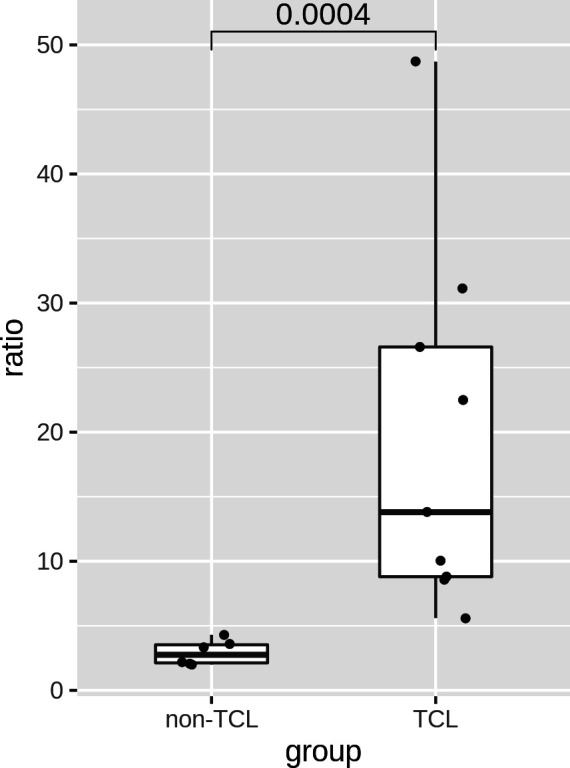
Ratios of the J2-2P~J2-3 to the J2-3 sequences in the BM samples of six non-TCL and six TCL patients probed by digital PCR. A p-value of Wilcoxon rank test is shown.

The ratios of TCL patients were significantly (p=0.0004) higher than those of non-TCL patients (**Figure 6**). A cutoff value of 5% would result in positive outcomes for all TCL patients, and negative outcomes for all non-TCL patients. Thus, the digital PCR assay of the J2-2P~J2-3 sequences retrieved five TCL cases with a negative BIOMED-2 test result. These results illustrate that the relative abundance of the J2-2P~J2-3 sequences could supplement the BIOMED-2 test.

## Discussion

To investigate the potential role of genetic background in the BIOMED-2 test, we adopted a 5’ RACE instead of a multiplex PCR approach to examine genetic sequences in the BIOMED-2 primer sites. In multiplex PCR, when a primer sequence differs from a genetic variant in the binding site, it can be the base of the primer that is sequenced, instead of the genetic variant. Such errors can be avoided in 5’ RACE. Besides, 5’ RACE probes RNA whereas the multiple BIOMED-2 primers inspect DNA (27). Thus, the distinct target of 5’ RACE has a good chance to supplement the BIOMED-2 test. NGS-based clonality test can improve the sensitivity of TCL diagnosis (28). However, the improvement is only moderate because the test focused on the same targets. In fact, this improvement was likely attributed to the different primers used for NGS. Another advantage of 5’ RACE is that it probes both recombined and NR sequences, the latter of which will be missed by multiplex PCR. Using NR reads to supplement TCL diagnosis is promising because NR sequences likely arise *via* a different mechanism from the recombined sequences, which are the targets of the BIOMED-2 test.

Although our NGS reads did not cover all primer sites in the V and J genes, we expected that the V and J sequences of clonal T cells, if present, would be abundant in the 5’ RACE data. Therefore, although the identified SNPs were not comprehensive, they should cover most of the V and J genes of the clonal TCRβ sequences. To expand coverage, we further examined the variants in dbSNP, which were more comprehensive. The TCRβ repertoire of the ALCL patient also confirmed that the absence of a TCRβ clonotype caused a negative test result. Putting together, it is unlikely that genetic background plays a role in the BIOMED-2 test for Taiwanese individuals.

Our NGS reads also allowed us to examine the compositions of various TCRβ clonotypes (i.e., repertoire) and types of NR TCRβ sequences. In addition to CR, PR, and NR reads, our analysis revealed NS, AS, and AR reads. Among these, we found that aberrant splicing was relatively common in the 5’ RACE data of the TCRβ gene, and the proportion of AS reads was even larger than PR reads in 21 of the 24 samples. We emphasize that AS reads can be derived from the transcripts of CR, PR, or NR genes. For example, V-C reads can result from the aberrant splicing of CR transcripts, whereas I-J-C reads can result from PR or NR transcripts. Thus, recombination and splicing occur at different levels and aberrant splicing does not necessarily reveal the recombination status of the TCR gene. From this viewpoint, our AS or even NR category is more descriptive than indicating the underlying gene recombination. To distinguish the difference, we used “read” or “sequence” for the observed RNA data, and “gene” to describe recombination status and the DNA equivalence, e.g., BIOMED-2 result and consensus of RNA sequences.

Approximately half of our 5’ RACE reads were NR sequences, which are largely overlooked in the literature, perhaps because they do not code for productive proteins. To our knowledge, only Fang et al. (2014) described NR sequences, which were called “unmapped-J-C” reads in their study (15). Interestingly, the authors found a higher percentage of unmapped-J-C reads in lung cancer patients than in healthy individuals. This suggests that NR sequences may serve as a biomarker of lung cancer. Here, we showed that NR sequences might supplement the TCL diagnosis. These studies suggest that NR TCR sequences should no longer be considered as a waste.

Fang et al. (2014) suggested that NR reads are the results of aberrant splicing, that is, a C gene segment splicing to a J gene segment downstream of the recombined J gene segment. This implies that aberrant splicing occurred in the CR or PR transcripts. The transcription of CR and PR genes is reasonable because the promoters upstream of the V and D gene segments on the TCRβ gene have been reported (29–31). More experiments are needed to elucidate the generation mechanism of the NR sequences.

The results of the BIOMED-2 test and digital PCR showed the importance of TCL subtypes and specific TCR genes. Our AITL and ALCL patients showed positive and negative BIOMED-2 test results. In a European cohort (11), the sensitivity of the BIOMED-2 test in AITL cases was also higher than that in ALCL cases (96% vs 79%). Besides, the BIOMED-2 tests of our PTCL-NOS patients revealed that TCRγ clonality was more prevalent than TCRβ clonality. This is consistent with the sensitivities based on the TCRγ and TCRβ genes (85% vs 54%) in our prior study (12).

For digital PCR, four and five TCL patients had positive and negative BIOMED-2 test outcomes, respectively. Despite their different test results, all TCL patients showed a higher relative abundance of the J2-2P~J2-3 sequences (5.6-48.7%) than the non-TCL controls (2.0-4.3%). This seems to contradict the result of NGS data, where the BIOMED-2 positive AITL patient did not show a high frequency of the J2-2P~J2-3 sequences. This disagreement can be explained by distinguishing between TCRγ and TCRβ clonality. In both the NGS data and digital PCR assay, only TCRβ sequences were examined. The AITL patient showed a monoclonal TCRβ pattern in the BIOMED-2 test. In contrast, all nine TCL patients for digital PCR showed a polyclonal TCRβ pattern. Because of their different TCRβ clonality results, it is reasonable that the fractions of the J2-2P~J2-3 sequences in the AITL patient and the nine TCL patients were different.

This study has some limitations. First, the samples from nine TCL and six non-TCL patients were not large. However, the difference between the TCL and non-TCL groups was substantial enough to achieve statistical significance using the small number of samples. With this result, we concluded that the J2-2P~J2-3 sequences may assist the BIOMED-2 test. The power of this potential biomarker for TCL diagnosis needs to be assessed in a large cohort in the future. Second, the quality control of RNA samples for digital PCR can be improved. A successful PCR requires long RNA fragments containing the primer-binding sites. The current quality control is a qualitative instead of a quantitative measure, which may introduce noise into the ratio. Third, the sequence composition of the NGS data may not truthfully represent a real sample because shorter sequences are amplified more efficiently in NGS and NR sequences are usually shorter than CR reads (32). Therefore, the ratios derived from NGS data and the digital PCR assay are not directly comparable.

NR TCR sequences are present in healthy individuals and TCL patients but have been overlooked in the literature. This is the first study to characterize NR TCRβ sequences in detail and shows their capability to supplement current TCL diagnosis.

## Data availability statement

The datasets presented in this study can be found in online repositories. The names of the repository/repositories and accession number(s) can be found below: BioProject, accession number PRJNA869856.

## Ethics statement

The studies involving human participants were reviewed and approved by Clinical Trial and Research Ethical Committee, National Cheng Kung University Hospital (IRB No. IRB-99-106, BR-100-137). Written informed consent for participation was not required for this study in accordance with the national legislation and the institutional requirements.

## Author contributions

Y-LC, C-LH, WH, and TL conceived and designed the experiments. Y-LC, W-LC, Y-HH, P-JC, and J-RC performed the experiments. CC, Y-TH, and T-YC contributed materials of T-cell lymphoma. TL and C-YH analyzed the data and Y-LC, N-HC, and TL wrote the paper. All authors contributed to the article and approved the submitted version.
