# Supplementary material for: Enhancing diagnosis of T-cell lymphoma using non-recombined T-cell receptor sequences
Source: Front Oncol. 2022 Dec 8;12:1014132. doi: 10.3389/fonc.2022.1014132 (PMC9772823; doi:10.3389/fonc.2022.1014132)
Supplement: Supplementary file 1 [file DataSheet_1.pdf]

Supplementary Materials

Supplementary Tables

Individual	Sex	Age	Note
I1	M	30-35	
I2	F	25-30	
I3	F	25-30	
I4	F	30-35	pregnant
I5	M	40-45	
I6	M	20-25	
I7	F	20-25	
I8	F	25-30	
I9	F	25-30	
I10	F	30-35	
I11	F	60-70	ALCL; BM before CT (b); PBs after CT (a)
I12	F	25-30	
I13	M	65-70	AITL
I14	M	45-50	
I15	M	35-40	Cold
I16	M	25-30	
I17	M	25-30	
I18	F	20-25	
I19	F	20-25	
I20	F	20-25	
I21	F	20-25	
I22	F	20-25	
I23	F	40-45	

Supplementary Table 1. Basic demography of 23 individuals providing samples for NGS. ALCL: anaplastic large cell lymphoma. BM: bone marrow. CT: chemotherapy. PB: peripheral blood. AITL: angioimmunoblastic T cell lymphoma.

Sample	de-multiplexed	Processed	Annotated	Chimeric	Uncertain
I1	4724	1421	1421	36 (2.5%)	2 (0.1%)
I2	5009	1094	1094	27 (2.5%)	1 (0.1%)
I3	4189	919	919	28 (3.0%)	4 (0.4%)
I4	19248	3923	3923	161 (4.1%)	51 (1.3%)
I5	4159	1162	1162	50 (4.3%)	2 (0.2%)
I6	118781	18090	18074	547 (3.0%)	44 (0.2%)
I7	6175	1175	1175	60 (5.1%)	2 (0.2%)
I8	4778	1035	1032	29 (2.8%)	6 (0.6%)
I9	5410	2186	2186	47 (2.2%)	4 (0.2%)
I10	10458	4661	4660	118 (2.5%)	3 (0.1%)
I11b	25971	12870	12868	57 (0.4%)	10 (0.1%)
I11a	20720	12425	12421	61 (0.5%)	8 (0.1%)
I12	33357	7612	7607	294 (3.9%)	125 (1.6%)
I13	11510	3480	3480	42 (1.2%)	13 (0.4%)
I14	23547	5985	5980	150 (2.5%)	27 (0.5%)
I15	27492	8338	8331	219 (2.6%)	55 (0.7%)
I16	23554	6164	6161	138 (2.2%)	28 (0.5%)
I17	4109	2341	2340	42 (1.8%)	3 (0.1%)

I18	8219	2509	2508	104 (4.1%)	6 (0.2%)
I19	4954	1823	1821	92 (5.1%)	5 (0.3%)
I20	1207	205	205	12 (5.9%)	1 (0.5%)
I21	2470	434	434	31 (7.1%)	1 (0.2%)
I22	6682	1352	1351	124 (9.2%)	28 (2.1%)
I23	3441	750	750	85 (11.3%)	16 (2.1%)
Total	380164	101954	101903	2554 (2.5%)	445 (0.4%)

Supplementary Table 2. Statistics of the TCRβ reads of all samples for NGS.

locus	reference base	variant base	gene	primer site	found previously	homo- or hetero-zygosity and the sample
22536	C	A	TRBI	N	N	he:I10
29742	T	C	TRBV6-1	N	N	he:I16,I18
75976	C	G	TRBV7-2	N	Y	ho:I6,I9,I10,I12|he:I2,I13,I15,I16,I17,I18,I19
76002	T	A	TRBV7-2	N	N	he:I6
76077	G	A	TRBV7-2	N	Y	ho:I1,I6,I9,I10,I12|he:I2,I13,I15,I16,I17,I18,I19
107959	G	T	TRBV7-3	N	N	he:I6
107968	G	A	TRBV7-3	N	Y	ho:I17|he:I11a
157496	G	A	TRBV11-2	N	Y	ho:I12,I17,I18|he:I6,I10
174563	T	C	TRBV6-5	N	N	he:I16,I18
192955	A	G	TRBV6-6	N	Y	he:I10
193053	T	C	TRBV6-6	N	Y	he:I10
205947	A	C	TRBV5-5	N	Y	ho:I6
215325	T	G	TRBI	N	N	he:I15
215335	G	A	TRBI	N	Y	he:I15,I16
230565	G	A	TRBV6-8	N	N	ho:I6
235271	C	T	TRBV7-7	N	N	ho:I6
272782	A	G	TRBV7-9	N	Y	ho:I1,I9,I10,I11b,I11a,I13|he:I6,I12,I14,I15,I17,I18,I19
272783	A	C	TRBV7-9	N	N	he:I6,I18,I19
279152	A	G	TRBV13	N	N	ho:I6
287672	A	G	TRBV10-3	N	Y	he:I12,I13,I14,I18
287746	C	T	TRBV10-3	N	Y	ho:I1,I6,I9,I10,I11b,I11a,I15,I16|he:I13,I14,I17,I18
287767	T	C	TRBV10-3	Y	Y	he:I12,I13,I14,I18
287809	G	A	TRBV10-3	N	Y	he:I12,I13,I14,I18
307089	A	G	TRBV12-4	N	Y	ho:I12|he:I2,I9,I13,I14,I17
307328	C	T	TRBV12-4	N	N	he:I2,I9,I12,I13,I14
336731	A	G	TRBV15	N	Y	ho:I6,I12,I13,I14,I15,I16
359002	G	C	TRBV18	N	Y	he:I12,I17
362286	T	C	TRBV19	N	Y	ho:I17,I19
362462	A	G	TRBV19	N	N	ho:I19|he:I18
362493	G	T	TRBV19	N	N	he:I16,I18
370327	T	A	TRBV20-1	N	Y	ho:I1,I12|he:I2,I3,I7,I13,I14,I17,I18,I19,I22,I23
370423	C	A	TRBV20-1	N	Y	ho:I1|he:I12,I14,I18,I19,I22,I23
370589	G	C	TRBV20-1	N	N	he:I13
398803	G	A	TRBV24-1	N	Y	he:I12,I17
412894	T	A	TRBV25-1	N	N	he:I6
457459	G	T	TRBI	N	N	he:I9
551517	C	T	TRBJ1-6	N	N	ho:I4,I6,I11a,I12,I14,I15|he:I10,I13,I16
559158	C	G	TRBI	N	N	he:I11a,I14,I15
559421	C	G	TRBI	N	N	he:I10,I13
559433	G	A	TRBI	N	N	he:I4,I13
559971	T	G	TRBJ2-7	N	N	he:I6,I7,I14
563337	T	C	TRBI	N	N	he:I6
563501	C	T	TRBI	N	N	ho:I11b,I11a,I14,I15|he:I6,I12
575122	A	G	TRBV30	N	Y	he:I6

Supplementary Table 3. Distinct SNPs of all samples on the TCRβ reference.

gene: V10-3 (287764..287788); primer: (Vbeta12a/3/13a/15)
SNP: 287767, T->C

```
5’>3’
CTATATGTACTGGTATCGACAAGAC
.||.|||||||||||||||||||||
ATACATGTACTGGTATCGACAAGAC
```

Supplementary Table 4. A SNP occurring in the binding site of a BIOMED-2 primer. The upper and lower segments are for the TCRβ reference and primer, respectively.

locus	dbSNP accession	ref_base> alt_base	alt_allele frequency	V/J gene	distance to 3’ end of primer	match status (ref v.s. alt)
9857	rs376217909	A>G	0.167	TRBV3-1	20	mismatch v.s. match
112633	rs781570489	C>A	0.218	TRBV5-3	13	match v.s. mismatch
131097	rs361466	C>T	0.01	TRBV11-1	12	match v.s. mismatch
148416	rs778495653	C>T	0.0004	TRBV10-2	7	match v.s. mismatch
174411	rs368420989	C>A	0.20	TRBV6-5	16	mismatch v.s. match
178585	rs1350743847	A>T	0.01	TRBV7-4	19	match v.s. mismatch
178592	rs763625518	A>T	0.02	TRBV7-4	12	match v.s. mismatch
178601	rs765065295	G>A	0.018	TRBV7-4	3	match v.s. mismatch
192981	rs555423746	G>A	0.008	TRBV6-6	18	match v.s. mismatch
215638	rs370378187	A>T	0.50	TRBV7-6	12	match v.s. mismatch
215647	rs375037927	G>A	0.50	TRBV7-6	3	match v.s. mismatch
243522	rs376369262	C>A	0.46	TRBV5-7	13	match v.s. mismatch
279395	rs915389856	C>T	0.01	TRBV13	11	match v.s. mismatch
287767	rs17246	T>C	0.26	TRBV10-3	21	mismatch v.s. match
298414	rs17272	T>G	0.05	TRBV11-3	4	mismatch v.s. match
304010	rs1163785552	C>A	0.02	TRBV12-3	21	match v.s. mismatch
341559	rs17284	T>G	0.01	TRBV16	18	mismatch v.s. mismatch
463071	rs370429371	C>T	0.002	TRBV28	8	match v.s. mismatch
549557	rs118195859	G>A	0.08	TRBJ1-2	13	match v.s. mismatch
560005	rs530526216	A>G	0.009	TRBJ2-7	20	match v.s. mismatch
575259	rs17267	G>A	0.05	TRBV30	16	match v.s. mismatch

Supplementary Table 5. SNPs in the binding sites of BIOMED-2 primers in the NCBI dbSNP database.

locus	confidence	Segment around donor site	VDJ
129139	1.00	CAGGAAGCAG^GTGAGGCCCA	NA
462820	1.00	CTGGCTGTAG^GTGAGTCCTG	TRBV28
494139	1.00	AGCTCTGGCG^GTGAGTGGGA	NA
554579	1.00	GGTAGAGCAG^GTGAGTGGGG	TRBC1
555297	1.00	GATGGCCATG^GTAAGCAGGA	NA
563925	1.00	GGTAGAGCAG^GTGAGTGGGG	TRBC2
492826	0.99	GCTACAAGTC^GTAAGTGTGG	NA
505058	0.99	AGCTCTGGTG^GTGAGTGGGA	NA
514331	0.99	GCTACAAGCC^GTAAGTGTGG	NA
525735	0.99	AGTTCTGGCG^GTGAGTGGGA	NA
534698	0.99	GCTACAAGTC^GTAAGTGTGG	NA
536009	0.99	AGTTCTGGTG^GTGAGTGGGA	NA

548538	0.99	GTGCCTGGAG^GTGAGAAGGA	NA
558913	0.97	ACCGTGCTAG^GTAAGAAGGG	TRBJ2-1
564709	0.97	GATGGCCATG^GTAAGGAGGA	NA
559394	0.95	ACAGTGCTCG^GTAAGCGGGG	TRBJ2-3
559665	0.95	CTGGTGCTCG^GTGAGCGCGG	TRBJ2-5
338660	0.94	CCGAGAGTGG^GTACGTGTGG	NA
564682	0.94	TGCCGTGCTG^GTCAGTGCCC	NA
268707	0.93	TACTAGAGAG^GTAAGTGGCT	NA
306793	0.93	AAGCTCCTAG^GTAAGGCATG	NA
312783	0.93	ATGAAACGGG^GTGAGTGAAT	NA
456596	0.93	GAGCTGGCTG^GTAAGGCTCT	NA
491636	0.93	GCAGCTGCTC^GTGAGTATCA	NA
503745	0.93	GCTACAAGTA^GTAAGTGTGG	NA
511053	0.93	ATGTGGCAAG^GTAAGGCTCT	NA
513142	0.93	GGAGCTGCTG^GTGAGTTTCA	NA
545538	0.93	ACCCCATGAG^GTAGGTATTA	NA
359561	0.92	GATCCAGCAG^GTAGTGCGAG	NA
559546	0.92	TCAGTGCTGG^GTAAGCTGGG	TRBJ2-4
208999	0.91	AGCACTGAAG^GTAAAAGTAT	NA
287954	0.91	TGTGCCATCA^GTGAGTCCAC	NA
533760	0.91	GGGAGACCAG^GTGGGGCTGG	NA
70120	0.90	ACACGGGGAG^GTGAGCAGTA	NA
96757	0.90	AGGAGGTCAG^GTCAGTCTTG	NA
60371	0.89	CTGCAAGCAG^GTGAGTCCTG	TRBV3-2
148590	0.89	TGCGCCAGCA^GTGAGTCCAC	NA
446591	0.89	ACAACTTTGG^GTAAGTGGTG	NA
558943	0.89	GGGAGAGAGG^GTGAGCAGCC	NA
50638	0.88	TACTCAGTTG^GTGAGGGTAC	NA
72319	0.88	TACTCAGTTG^GTGAGGGTAC	NA

Supplementary Table 6. Predicted splice donor sites on the plus strand of the TCRβ reference and the corresponding VJ genes.

Supplementary Figures

A
BM:

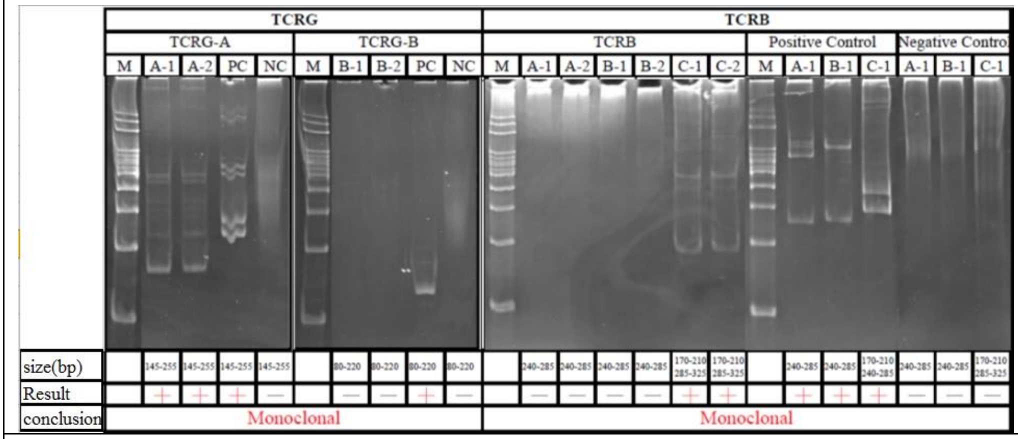

B
BM:

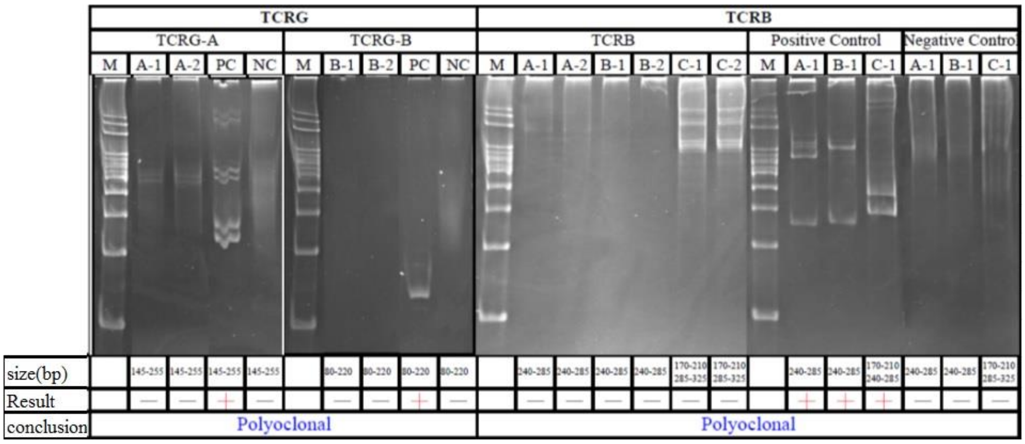

PB:

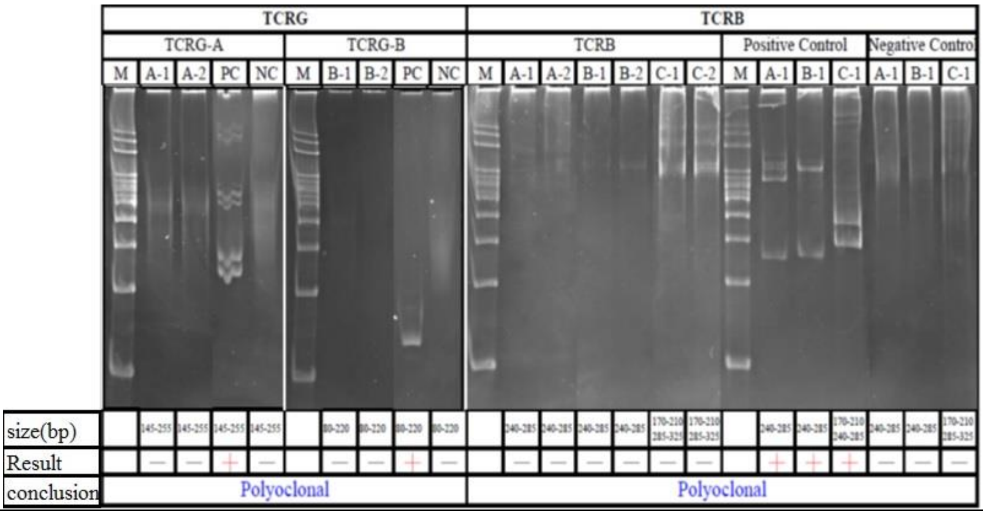

Supplementary Figure 1. BIOMED-2 clonality test results based on TCRγ and TCRβ genes in the bone marrow (BM) and/or peripheral blood (PB) samples of the (A) AITL and (B) ALCL patients. PC: positive control; NC: negative control.

The BIOMED-2 test on the peripheral blood (PB) sample of the ALCL patient is also performed, and the result is consistent with that of the BM sample.

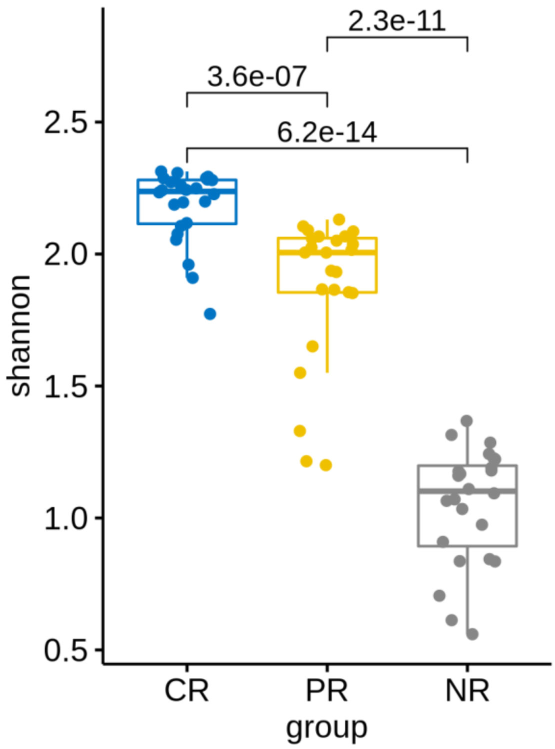

Supplementary Figure 2. Shannon diversities based on J-gene compositions in CR, PR, and NR reads for all samples. Wilcoxon-test p-values between groups are shown.

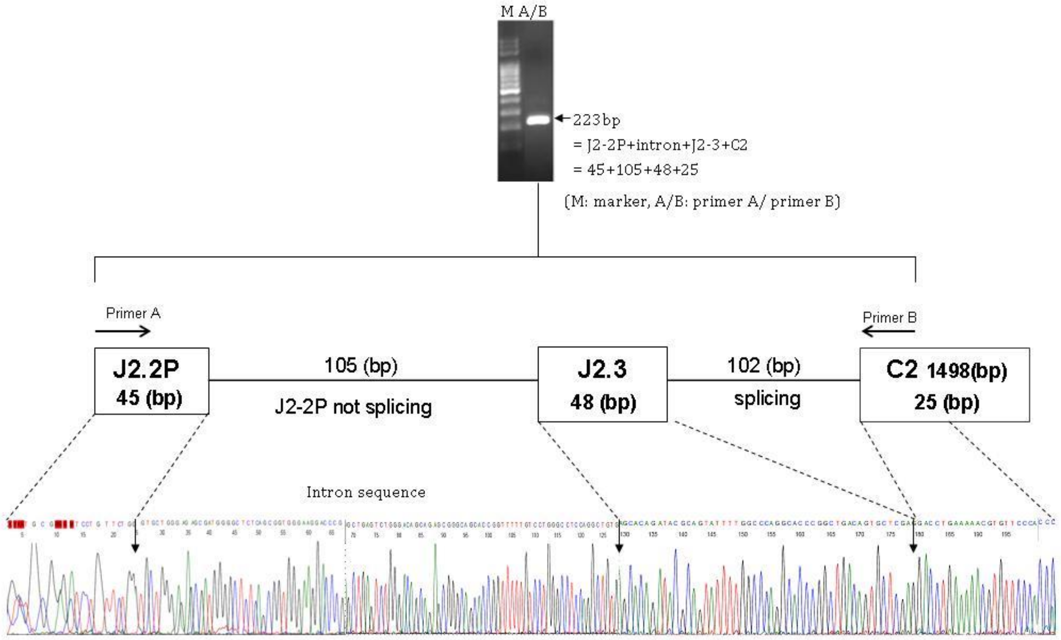

Supplementary Figure 3. Validation of the J2-2P~J2-3 sequence via amplification and Sanger sequencing. The TCRβ segments from J2-2P to C region are amplified using primer A (J2-2P): 5'-ACCCTGTTCTTAGGGGAGTG-3' and primer B (TCRβ-C2): 5'-CACAGCGGCCGCGGGTGGGAACACGTTTTTCAGGT-3'.

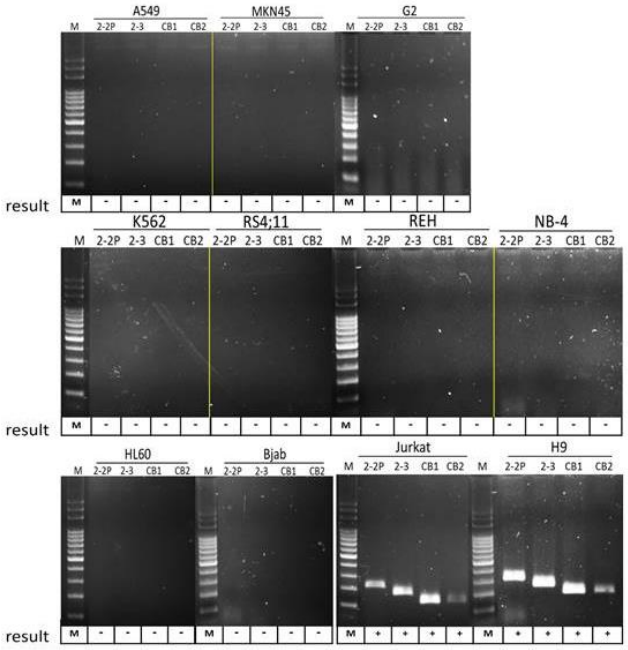

Cell line	Types	Disease
A549	Solid Tumor	Lung cancer
MKN45	Solid Tumor	Gastric adenocarcinoma
G2	Solid Tumor	Hepatocellular carcinoma
K562	leukemia/Fusion	CML; bcr-abl
RS4;11	leukemia/Fusion	ALL; KMT2A-AFF1
REH	leukemia/Fusion	Acute lymphocytic leukemia
NB-4	leukemia/Fusion	APL
HL60	leukemia	APL
BJAB	B Cell lymphoma	Burkitt lymphoma
Jurkat	leukemia	Acute T cell leukemia
H9	T Cell Lymphoma	Cutaneous T lymphocyte

Supplementary Figure 4. Detection of the J2-2P~J2-3 and the J2-3 transcripts in the cell line panel by RT-PCR. The J2-2P-J2-3 and J2-3 transcripts are detected only in cell lines of T-cell lineage (Jurkat and H9), as compared with solid tumor cell lines. J2-2P, J2-3, CB1, and CB2 are TCRβ specific primers (see main text).
